# Dr. J. Henry Fruitnight

**Published:** 1901-02

**Authors:** 


Dr. J. Henry Fruitnight, of New York, died at his home in that city December 18, 1900, aged 49 years. He was an alumnus of Bellevue Hospital Medical College, 1875. He was a pediatrician of note, a member of the American Pediatric Society, and a frequent contributor to the the literature of that branch of medicine. He did not believe, evidently, that the title of "professor" was the goal of a physician's ambition, for he was great enough to have refused two offers of that kind, on the ground that teaching might appropriate time that rightfully belonged to his patients.

